# The Finnish Pension System and Its Future Challenges

**DOI:** 10.1007/s10272-020-0877-1

**Published:** 2020-04-01

**Authors:** Tarmo Valkonen

**Affiliations:** ETLA Economic Research, Arkadiankatu 23 B, 00100 Helsinki, Finland

## Abstract

A specific feature in the Finnish pension system is rule-based preparation for mortality change. The earned pension capital is adjusted to life expectancy and the lowest age limit of the flexible retirement age will be adapted so that the ratio of expected years in employment and retirement is fixed after year 2030.

The Finnish pension system has succeeded in gaining high social, and reasonable financial sustainability. The balance between reaching the ambitious redistribution goals and minimising labour supply distortions is achieved with tax-financed, income tested basic pensions, income-tested basic pensions and a strong link between wage income and accrued pensions for middle- and high-income workers in the earnings-related schemes. These well-governed first-pillar schemes have high coverage and similar benefit rules. Second pillar occupational pensions are rare in Finland.

One of the secrets of its success has been the capacity to make extensive reforms when required. By law, the earnings-related pension scheme follows the defined benefit rule, where contribution rates adjust to shocks that weaken the contribution base or increase expenditures. In practice, however, an outlook of a strongly increasing contribution rate has often triggered a reform process. Both the negotiations and the full implementation of the reforms have taken time, but the outcomes have been largely accepted.

A specific feature in the Finnish pension system is rule-based preparation for mortality change. The earned pension capital is adjusted to life expectancy and the lowest age limit of the flexible retirement age will be adapted so that the ratio of expected years in employment and retirement is fixed after year 2030. Postponed withdrawal of pensions is rewarded in an actuarially fair way. This set of rules generates strong incentives to extend working life when life expectancy increases. Hence, the rules promote adequacy of pensions and fiscal sustainability.

Another non-standard element is the partial pre-funding of the first-pillar earnings-related benefits. The share of contributions that is pre-funded in the dominating private sector pension scheme (TyEL) is small, but the required return on funds is low, and the amount of excess is saved to support the funding. The pension scheme is run by private pension companies and other pension institutes, which are individually liable for the pre-funded part of the pensions, but mutually responsible for financing the pay-as-you-go part. Public sector schemes have buffer funds that aim to smooth contribution rates. As an outcome, about a third of the accrued earnings-related pension rights are pre-funded.

The main challenge of the pension system is population ageing, which is escalating in Finland due to the recent large fall in fertility. If the number of children remains low and net migration does not increase markedly, there will be a growing need to increase contribution rates. Moreover, the life expectancy adjustment of pensions lowers replacement rates if mortality rates fall as expected. It will be compensated partly by longer working careers, but full compensation would require a faster-than-projected increase in employment rates. Other risks that may influence the sustainability of the pension system include lower growth in employment and wages due to technological development as well as lower pension fund yields than expected.

This review first provides a brief outlook on the various development stages of the Finnish pension system that are still visible in its basic structure and the logic of the current version. The subsequent section illustrates with some key indicators how the current system has succeeded in reaching its goals and shows its risk-sharing properties. This is followed by a discussion of how the pension system is prepared to cope with future challenges that are common to pension schemes across industrialised countries. The concluding section suggests changes that could make the Finnish system even more resilient.

## From fragmental benefits of the employed to universal and uniform social security

The history of Finnish old age pensions dates to pensions of the civil servants in 19th century, but a first scheme with substantial coverage was established in 1939, when the national pension system was introduced. This earnings-related scheme was based on fully funded individual accounts. The negative experiences of war-time inflation and political resistance led to the 1957 pension reform that included abandonment of pre-funding, equalisation of pensions, introduction of income- and wealth-tested

**Tarmo Valkonen**, ETLA Economic Research, Helsinki, Finland.

supplementary allowance and tax financing. In 1996, the basic part of the pension was abolished and only the income-tested part of the national pension remained. The guarantee pension, which defines the minimum amount of pension income, was introduced in 2011. Disability pensions were part of the system from the beginning.

The general earnings-related private sector pension scheme was introduced in 1962. While based on law, it was an outcome of negotiations between labour market parties. Since then, the social partners have had a decisive role in the preparation of pension reforms and strong representation in the governing bodies of the pension funds. Different sectors prepared their own laws during the 1960s and ended up with a different set of rules. The low retirement ages and high accrual rates of the public sector schemes particularly stuck out. The private sector schemes and the national pension scheme had a retirement age of 65 years.

During the next two decades, benefits were expanded. In the 1970s, the accrual rates were raised markedly, and the initially low pensions were topped up with discretionary increases. The unemployment pension was introduced for the long-term unemployed who were at least 60 years of age. The eligibility age for these pensions was lowered to 55 years in the 1980s. Several new early retirement schemes were introduced in 1986. The popularity of early retirement surprised policy planners.

The deep recession in the beginning of the 1990s and the continuous increase in life expectancy initiated a period of retrenchments. First, the liability to pay pension contributions, previously solely on employers, was partially shifted to employees. More importantly, it was agreed that the future increases in contributions are distributed on a 50/50 basis. The true incidence of the hikes in the employers' contribution rates had always been mainly on wages because they were agreed by the central labour market parties, but this reform further strengthened the responsibilities of the labour unions. Also, a long process of limiting access to early retirement schemes started.

The investment policies of pension funds changed radically during the 1990s. In times of undeveloped financial markets, pension wealth was an important source of liquidity and investment funding for domestic firms. The real rate of return on pension funds was often negative due to a high inflation rate and the use of investment income exceeding the required return to lower contributions. The development of the financial markets and the alleviation of investment regulations of the funds promoted by striving for higher returns and more diversification rapidly increased the investments in stock and foreign markets. The public sector pension institutes started buffer-type pre-funding of the contributions in the late 1990s.

An extensive and radical pension reform took place in 2005. The reformed rules form the backbone of the current pension system. Key elements were the harmonisation of the benefit rules of different earnings-related schemes, a tighter link between earned income and accruals, introduction of flexible old age retirement, gradual abolishment of several early retirement schemes and the introduction of a link between life expectancy of the retiring cohort and the pensions. Pension accrual starts from an earlier age and smaller amounts of wages are counted. A pensionable wage is determined by the whole working career. Pensions are fully portable allowing job and sector changes without losses in accrued amounts. The maximum replacement rate was abolished. The accrued pension rights are indexed to average wages and consumer prices with weights 80/20 during working years. For the pensions in payment, the ratio is 20/80. Some pensions accrue also during periods of unemployment, child care, sick leave and studies.

The possibility to withdraw the pension was separated from the decision of retiring from work. The only remaining link was that postponement of pension withdrawal was rewarded with a higher accrual rate only when working life continued. Flexible retirement allowed retirement between the ages 62 and 67, but withdrawal at age 62 caused a marked loss in the pension if the person was not unemployed long term. One of the ideas behind the flexible retirement age was that the expected future decline in pensions due to increasing longevity could be compensated by extending voluntarily working careers. It turned out, however, that old age retirement concentrated at age 63 and there was the risk of a continuously falling replacement rate.

The observed reluctance to extend voluntarily working lives and a continued rise in projected life expectancy generated the need for a new pension reform. It was understood that the low retirement age endangered both adequacy of pensions and financial sustainability of the general government. The reform, implemented in 2017, introduced a stepwise increase in the lower age limit of the flexible pension age until it reaches age 65 in 2027 and establishes a link between life expectancy and the lowest retirement age in 2030. The link is calibrated so that for each additional year of life expectancy, the lower age limit goes up by eight months. The link is also applied to basic pensions and early retirement pensions except disability pensions. The upper age limit of the flexible retirement age will be raised from 68 to 70 years.

The higher accrual rate earned from work after the lowest retirement age was replaced by a reward for postponing withdrawal of the pension. The reform also included two minor changes in early retirement schemes. The generous part-time pension scheme was replaced by the actuarially fair possibility of drawing part of the old age pension from age 61. The second new element is a years-of-service pension, which can be drawn from age 63 if a stressful working career has continued for at least 38 years and the working capacity of the individual has declined. The years-of-service pension remains unpopular because access to benefits is complicated and uncertain and it is smaller than the disability pension. The goal of higher employment periods near retirement has been supported by shortening the period of earnings-related unemployment benefits paid before reaching the lowest eligibility age for old age pensions.

Private sector pensions are financed by contributions collected from employers and employees. Contributions are deductible in income taxation, pensions are taxable, and there is no tax on the yields of the funds during the savings period (exempt-exempt-taxed principle). Entrepreneurs have similar benefit rules, but they have flexibility in declaring the amount of labour income and thereby can influence the paid contributions and accrued benefits. In addition, government supports their pensions.

The key features of the current earnings-related pension system are universality and uniformity. Policy changes are effective, because the rules apply to almost everyone, and the first-pillar benefits cover a large share of the incomes of the retired population. For those whose earnings-related pensions are small, the pensions-tested tax-financed national pension tops up the income. Guarantee pensions ensure the minimum level of income. Low-income pensioners are also eligible for a housing allowance.

### How does the Finnish pension system perform?

The assessment of the performance of the pension system requires a characterisation of its goals. In an agreement on the main features of the 2017 pension reform, the social partners set a smooth development of the contribution rates, long-term protection of the benefits and ensured financing as the targets (Social partners, 2014). This statement reflects the reality well: even though the system is based on the principle of defined benefits, the goals of constant contributions and fair burden sharing between generations have high priority.

The aims of the basic pensions are harder to clarify. National pensions and guaranteed pensions are indexed to consumer prices, which means that their development does not capture the growth of real wages. When the gap between purchasing power of basic pensions and earnings-related pensions became large enough, political reality required discretionary increases in the level of these pensions. The importance of the income-tested basic pensions has declined strongly during recent decades because the maturing of the earnings-related pension schemes has reduced the number of eligible pensioners with sufficiently small earnings-related pensions.

Pension income represents about 85% of all incomes of pensioners. The rest comprises mainly labour and capital income. The disposable income of pensioners is also influenced by strongly progressive taxation, which leaves pension income from national pensions untaxed. The poverty rate among pensioners is 13% (when the criterion is 60% of the median disposable income), which is the same as the average rate in the total population and somewhat lower than the EU average. Living standards of pensioners are also supported by the extensive underpriced public health and long-term care services.

The Finnish Centre for Pensions provides long-term projections on pensions, total expenditure and contribution rates (Tikanmäki et al., 2019). The outlook, based on the 2019 population projection of Statistics Finland, shows that the ratio of average total pensions to average wages is expected to decline by around ten percentage points in 65 years mainly because of the life expectancy adjustment of pensions.

The outlook of the financial sustainability of the earnings-related pension system strongly depends on the time period studied. The passing of the baby boomer generation starts to bring down the ratio of expenditures to GDP in the 2030s. Low fertility and extending longevity turn the trend when approaching the middle of the century. The main private sector pension scheme can keep the contribution rates stable until the 2050s, but after that there will be a strong and continuous increase. If the scheme aims to smooth development of contributions until 2085, it should raise the contribution rate immediately by 2.6 percentage points (Tikanmäki et al., 2019). This means that the scheme is not financially sustainable. In the public sector, the current very high costs are expected to converge in the long term towards the same level as in the private sector, which allows a minor decline in the ratio of contributions to wages.

The pension reforms, together with improved education and health of the retiring cohorts have increased the employment rates of elderly persons dramatically in Finland. The gain in the 60-64 age group has been more than 20 percentage points since the 2005 pension reform, which is almost three times higher than the improvement in the total employment rate. It is very likely that the growth will continue at a rapid pace due to the rising statutory retirement age.

The real rate of returns to pension funds has been reasonably high after the investment policies were liberalised (4% in 1997-2018). The low riskless interest rate has boosted asset values and risk premiums have grown, compensating the decline in interest income in recent years. The returns have been somewhat higher in the public sector buffer funds, where risk-taking is not restricted by solvency rules.

History shows that demographic development is difficult to project in the long term. As population projections are important for the sustainability of pay-as-you-go financed defined benefit pensions, it is useful to assess the uncertainty involved. Analysis performed using the stochastic population projections as inputs in an economic model shows that the Finnish idea of linking both pensions and the retirement age to life expectancy can manage longevity uncertainty very well. Longer working life helps to mitigate the replacement rate decline at the same time as the financial sustainability of both the pension system and the general government is improved (Lassila and Valkonen, 2018).

The other demographic and economic risks are carried by the contribution rate in Finland, at least if their realisation does not trigger pension reforms. The decreased contribution base requires that the contributions remain higher until the generations that have accrued less pension rights retire. Therefore, an observed reduction in the sum of wages and salaries is more problematic with a higher interest rate and lower future growth of contributions. Realisation of the risks related to the yield of the pension funds tend to increase contribution rates when the funds are used to pay pensions.

## Future challenges

The hottest topic of the current pension discussion in Finland is the rapid fall of the fertility rate during the past ten years. As there has been no decline in the amount of public resources used to support families by income transfers and services, and the employment rates of the young adults have improved, the reasons behind the declining number of births is not likely to be economic. Lack of information about the underlying causes means that there is also large uncertainty about the recovery of fertility and the efficiency of possible policies to promote it. The policy makers must consider the possibility that the fall in fertility weakens the finances of the pension scheme and general government permanently. Fertility risks are less important for schemes with individual or cohort-specific pre-funding. Another, more controversial possibility would be to add a link between the number of children raised and the amount of pension accrued at the individual level (Sinn, 2005).

Another trend that possibly weakens the tax and contribution bases is the potential fall in labour income caused by technological development. Robotisation and digitalisation influence the relative use of labour and capital in production, factor income shares and possibly also unemployment (Acemoglu et al., 2020). A related trend that is already observable in the Finnish labour market is the polarisation of jobs and wages. Even though there are large uncertainties about the future development of these trends, their potential influence on wages and pensions may be significant.

An essential issue in financial sustainability of a pay-as-you-go financed pension system is the link between labour income and accrued pensions. If it is tight, a fall in the total wage bill also reduces pension accruals and finally pension expenditure. The challenge is to finance the pensions in payment during the time gap between the immediate realisation of lower contribution income and the future decline in paid pensions.

Third topic that has recently received renewed attention is the interaction of old age pensions and socio-economic differences in life expectancy. Well-educated people, who have higher wage income and larger pensions, live longer and benefit from old age pensions more. Moreover, an increase in the statutory retirement age means that the share of one's lifetime as pensioner declines more for the less educated. This is seen as unfair (Sánchez-Romero et al., 2019).

The average working careers of less-educated workers, on the other hand, end earlier, and there is often a period of disability or unemployment before old age retirement. Therefore, the realised redistribution depends strongly on the income available after early retirement compared to the old age pension. In the case of Finland, a simulation study showed that the average welfare of the less educated does not decline when the retirement age increases (Lassila et al., 2015). More generally, the main emphasis should be put on reducing the life expectancy differences instead of requiring poorly specified socio-economically adjusted retirement ages.

A fourth recent trend, which influences especially pre-funded pension schemes, is the low interest rate of low-risk government bonds. In pre-funded defined contribution schemes, the low yield means that the adequacy of pensions is at risk. In pre-funded defined benefit schemes, the contributions are increased either immediately due to solvency issues, or later when the funds are used to pay pensions. The Finnish private sector pension institutes have a unique solvency rule that allows a decline in the pre-funded share of the accrued pension liability when stock market prices fall. It has been suggested that this share should be enlarged to allow a riskier investment policy as a response to the low interest rates. This would improve the average return on the funds and mitigate potential problems because of solvency requirements (such as the forced sale of risky assets during recessions), but it increases the variation in the contribution rate, when the funds are used to pay pensions.

The COVID-19 crisis weakened the financial sustainability of the pension system markedly, but is not expected to result in an abandonment of the system's basic principles. The social partners reacted to the crisis by suggesting a temporary 2.6 percentage point cut in the employer's pension contribution rate. This was accepted by the government. The reduction will be compensated during the 2022-2025 period by higher contributions.

## Concluding remarks

The Finnish pension system has yet to solve some issues related to efficiency and risk sharing, yet it has many features that serve as an example of a well-defined and robust way to ensure old age security at reasonable costs.

The most urgent challenge is to share the fiscal consequences of lower fertility fairly between generations. The current pre-funding alleviates the problems somewhat, but the risk of a large jump in contribution rates is too high. A rules-based way of improving intergenerational fairness would be to establish a link between the pre-funding rate and the fertility rate. A more precise instrument would be a link between the accrual of pensions and the fertility rate of a cohort.

There is some room for diminishing pension expenditure by removing poorly justified elements. Accrual of earnings-related old age pensions from periods when the individual does not pay contributions is one of them. It is the manifestation of a tendency to introduce redistribution in all parts of the tax and benefit system, also benefitting those who are well-off in terms of lifetime income. Another discretionary policy that would support overall financial sustainability would be to increase the lowest retirement age before implementing the link to longevity.

The main lesson to be learned from the history and the performance of the Finnish pension system is that resiliency can be achieved in two ways. One is a preparedness to make pension reforms, whenever financial or social sustainability are at risk. Another is to agree beforehand on rules that redistribute the outcomes of the risks in a way that is acceptable.

In Finland, the increased weight on financial sustainability and intergenerational fairness in the decision-making of the social partners has enabled implementation of balanced reforms, where excessively generous early retirement schemes have been abolished and the link between the wages earned and pensions accrued is strengthened. A key feature is also the new rules that promote longer working lives as a response to increased life expectancy.

In general, optimal risk sharing between generations is difficult to define. We observe the outcome of the interaction of current pension rules and realised demographic and economic risks but know little about the alternatives. One way of investigating risk sharing is to use stochastic simulations, which describe the outcomes of the current and alternative rules in hundreds of demographic and economic futures. This method provides a set of choices for policymakers.

If the pension system is reformed when required instead of investing in risk sharing rules, the system would benefit from a practice that triggers automatic adjustments to benefits and contributions until a reformed scheme takes effect. Such a rule would speed up the adjustments processes.
